# The Impact of Sleep on Haematological Parameters in Firefighters

**DOI:** 10.3390/clockssleep6030021

**Published:** 2024-06-26

**Authors:** Sara Alves, Francisca Silva, Filipa Esteves, Solange Costa, Klara Slezakova, Maria Alves, Maria Pereira, João Teixeira, Simone Morais, Adília Fernandes, Felisbina Queiroga, Josiana Vaz

**Affiliations:** 1 Instituto Politécnico de Bragança, Campus de Santa Apolónia, 5300-253 Bragança, Portugal; 2 Department of Veterinary Sciences, University of Trás-os-Montes and Alto Douro, 5000-801 Vila Real, Portugalfqueirog@gmail.com (F.Q.); 3 Environmental Health Department, National Institute of Health, Rua das Taipas 135, 4050-600 Porto, Portugal; filipa.esteves3@hotmail.com (F.E.); solange.costa2@gmail.com (S.C.); jpft12@gmail.com (J.T.); 4 EPIUnit, National Institute of Public Health, University of Porto, Rua das Taipas 135, 4050-600 Porto, Portugal; 5 REQUIMTE/LAQV, ISEP, Polytechnic of Porto, Rua Dr. António Bernardino de Almeida, 4249-015 Porto, Portugal; klaras@fe.up.pt (K.S.); sbm@isep.ipp.pt (S.M.); 6 AquaValor-Centro de Valorização e Transferência de Tecnologia da Água-Associação, Rua Dr. Júlio Martins n.º 1, 5400-342 Chaves, Portugal; maria.alves@ipb.pt; 7 Centro de Investigação de Montanha (CIMO), Instituto Politécnico de Bragança, Campus de Santa Apolónia, 5300-253 Bragança, Portugal; josiana@ipb.pt; 8 LEPABE-ALiCE, Faculdade de Engenharia da Universidade do Porto, Rua Dr. Roberto Frias, 4200-465 Porto, Portugal; 9 Research Centre for Active Living and Wellbeing (LiveWell), Instituto Politécnico de Bragança, Campus de Santa Apolónia, 5300-253 Bragança, Portugal; 10 Laboratório Associado para a Sustentabilidade e Tecnologia em Regiões de Montanha (SusTEC), Instituto Politécnico de Bragança, Campus de Santa Apolónia, 5300-253 Bragança, Portugal

**Keywords:** haemoglobin, haematocrit, PSQI, circadian rhythms

## Abstract

Sleep is a vital process that impacts biological functions such as cell renewal, bone regeneration, and immune system support. Disrupted sleep can interrupt erythropoiesis, leading to fewer red blood cells, reduced haemoglobin concentration, and decreased haematocrit levels, potentially contributing to haematological disorders. This is particularly concerning for shift workers for example firefighters. While previous studies have explored sleep’s adverse effects on various professions, research specific to firefighters is limited. This study investigates the relationship between sleep quality and haematological parameters among firefighters in Northeast Portugal. From a sample of 201 firefighters, variations in red blood cells, haemoglobin, and haematocrit values were linked to sleep quality. The study utilised non-parametric tests (Wilcoxon-Mann-Whitney, Spearman’s correlation) to explore the connection between sleep quality and haematological profile. The impact of covariates on haematological parameters was assessed using non-parametric ANCOVA (Quade’s). A multiple regression analysis was employed to further understand how sleep quality and various confounding variables impact haematological levels. Findings suggest a negative link between sleep quality and haematological levels, meaning that as sleep quality deteriorates, there is a tendency for haematological levels to decrease, as indicated by Spearman’s correlation (rRBC = −0.157, pRBC = 0.026; rHb = −0.158, pHb = 0.025; rHCT = −0.175, pHCT = 0.013). As observed in scientific literature, the correlation found suggests a possible inhibition of erythropoiesis, the process responsible for red blood cell production. Despite firefighters presenting a haematological profile within the reference range (RBC: 5.1 × 10^6^/mm^3^ (SD ± 0.4), Hb: 15.6 g/dL (SD ± 1.3), 47% (SD ± 1.0), there is already an observable trend towards lower levels. The analysis of co-variables did not reveal a significant impact of sleep quality on haematological levels. In conclusion, this study underscores the importance of sleep quality in determining haematological parameters among firefighters. Future research should investigate the underlying mechanisms and long-term implications of poor sleep quality on firefighter health. Exploring interventions to enhance sleep quality is vital for evidence-based strategies promoting firefighter well-being.

## 1. Introduction

Sleep is a fundamental process vital to maintaining individuals’ physical and mental well-being, promoting a higher quality of life [1].

It is during sleep that the body undergoes several essential functions, such as the continuous renewal and proliferation of cells [2], bone regeneration by promoting the proper proliferation of osteocalcin [3], and stimulation of the immune system through the synthesis of cytokine proteins, specifically interleukins and prostaglandins [4,5,6]. Additionally, studies suggest that sleep plays a crucial role in the process of haematopoiesis [7,8], which is the continuous lifelong process of production, growth, and differentiation of haematopoietic stem cells (HSCs) and haematopoietic progenitor cells (HSPCs) into mature specialised blood cells, such as leukocytes (white blood cells), thrombocytes (platelets), and erythrocytes (red blood cells) [9,10,11,12]. The process of formation of RBC (erythropoiesis) within the bone marrow occurs under the influence of erythropoietin (EPO) [13,14,15]. EPO’s primary purpose is to correctly maintain oxygen levels in the body [16] by stimulating the daily production of new RBC to compensate for its limited lifespan of 110–120 days [16,17]. While Hb concentration decreases, the amount of circulating EPO rises [17]. The release of erythropoietin is tightly affected by the oxygen levels in the body [18,19,20]. In response to situations of hypoxia or decreased oxygen supply to tissues [21,22], hypoxia-inducible transcription factors (HIFs) bind to hypoxia-response elements (HRE), activating EPO expression [23,24] and EPO synthesis and production by the kidney [21,22]. When erythropoietin is released into the bloodstream, it binds to specific receptors on erythroid progenitor cells in the bone marrow. This binding triggers a cascade of signalling events, leading to these cells’ proliferation, differentiation, and maturation into mature red blood cells (RBCs) [19,25]. Studies report EPO levels in the body follow a robust circadian rhythm [26], with a notable variation in Serum-EPO levels over the course of a 24-h day, reaching maximum levels at night (8 p.m. to 4 a.m.) [27] and lower levels in the early morning (4 a.m. to 8 a.m.) [28]. Researchers have conducted studies aiming to understand the genetic regulation of Epo in the kidney and its adherence to a circadian rhythm. Nevertheless, we were unable to identify a specific genetic element within the EPO gene responsible for directly governing this circadian fluctuation [28,29].

Prolonged sleep duration or quality changes can disrupt the body’s circadian rhythm, adversely affecting biological processes [30,31]. Poor sleep quality is a significant concern, especially for professions involving shift work or rotating schedules, which can result in sleep deprivation or disruption. Numerous studies have explored the detrimental effects of compromised sleep quality due to shift work, rotating schedules, sleep deprivation, and interruptions [32,33,34,35]. Studies realised with occupational groups such as police [36], military personnel [37], health professionals [38], and firefighters [39] concluded that abnormal functioning of the circadian rhythm is linked to metabolic changes and, in the long term, to the development of different types of pathologies and negative consequences for the health of these professionals [40].

While the scientific literature on the impact of sleep quality on firefighters is expanding, more is needed. Firefighters often face demanding work schedules, inadequate rest, and an increased risk of sleep deprivation. Further research is necessary to comprehend the full implications of sleep quality for their well-being. Additionally, the current scientific literature on the influence of sleep quality on blood cells, such as haemoglobin and haematocrit levels, is limited. So, investigating this potential association among firefighters becomes increasingly crucial.

In light of these gaps, our research question aims to address the following: What is the influence of sleep quality on the haematological profile of a group of firefighters in north-eastern Portugal? By exploring this specific population, we can gain valuable insights into the relationship between sleep quality and haematological indicators, contributing to a better understanding of the health implications for firefighters and informing targeted interventions.

## 2. Results

### 2.1. Participant Characteristics

A total sample of 233 firefighters from volunteer fire brigades of the district of Bragança, from the Northeast of Portugal, was obtained, of which only 201 (86.3%) have fully completed the PSQI questionnaire and have had blood sample collection. Out of the 233 participants, 36 (16%) were female, 170 (73%) were male, and 27 (12%) did not provide their sex, as can be seen in Table 1. Regarding age groups, 38% of participants were between 18 and 34, 46% were aged between 35 and 49, and 13% were above 50. The analysis of BMI parameters among the participants revealed striking findings, with only 27% of the individuals having a normal BMI, while a significant proportion, 49%, were classified as above weight. Despite this higher prevalence of overweight individuals, an encouraging 73% of the participants reported engaging in regular physical exercise. Furthermore, 40% of the sample consisted of smokers, and almost 25% of the participants admitted to having daily drinking habits. Additionally, 82% of the respondents reported regular consumption of coffee.

### 2.2. Quality of Sleep Assessment

Regarding the descriptive analysis of PSQI, resumed in Table 2, it is possible to conclude that 55% of the sample had good sleep quality, while 45% had poor sleep quality. Notably, the subjective sleep quality reported by each individual significantly differed from that indicated by the scale, as 89% claimed to have good sleep quality, while only 11% reported poor sleep quality. Such results are deeply concerning, as 45% of the sample experienced poor sleep quality and 28% slept for six hours or less each night.

### 2.3. Haematological Profile

The haematological parameters of interest (RBC, Hb, and HCT) were analysed to provide a descriptive understanding of the baseline values of the population in the study. The mean RBC level reported was 5.1 × 10^6^/mm^3^ (SD ± 0.4), comprising 4.9 ± 0.3 × 10^6^/mm^3^ for females and 5.4 ± 0.3 × 10^6^/mm^3^ for males. The mean Hb level was 15.6 g/dL (SD ± 1.3), with 13.9 ± 1.0 g/dL for females and 15.8 ± 0.7 g/dL for males. Similarly, the mean HCT was 47% (SD ± 1.0), with 44.8 ± 2.7% for females and 50.3 ± 2.8% for males. When comparing these values to the standard reference, it is essential to note that the regular RBC reference ranges from 3.85 to 5.20 × 10^6^/mm^3^ for females and 4.31 to 6.40 × 10^6^/mm^3^ for men [41]. Similarly, the mean Hb level falls within the normal range for adults, ranging from 13.6 to 18.0 g/dL for males and 11.5 to 16.0 g/dL for females. The normal HCT reference range for adults is typically between 39.8% and 52% for males and 34.7% and 46% for females. From a haematological perspective, the study population does not exhibit any significant abnormalities or deviations from the normal reference ranges, according to Direção-Geral de Saúde, Regulation nº 21/2008.

### 2.4. The Effect of Sleep Quality on Haematological Profile

**Figure 1:**
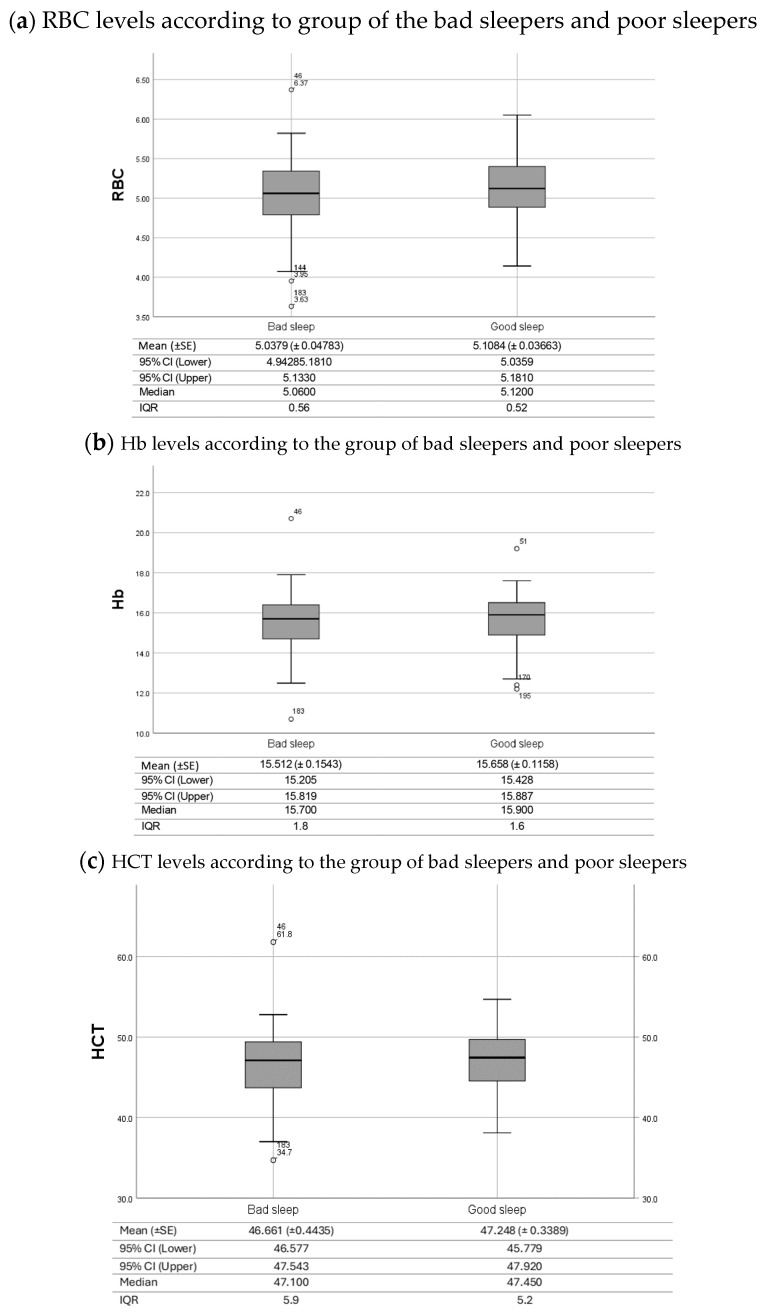
Box plot of haematological values ((**a**)—RBC, (**b**)—Hb, and (**c**)—HCT) of firefighters classified as having bad sleep quality and Good Sleep Quality. Horizontal lines indicate the median, boxes indicate the interquartile range, whiskers extend to upper adjacent values and lower adjacent values, and dots represent outliers. Note: The heamatological reference interval is indicated in the text.

The statistical relationship between sleep quality and haematological profile was examined, explicitly focusing on RBC, Hb, and HCT levels. By analysing the collected data, insights were obtained regarding the potential influence of sleep on these values among firefighters. A Spearman correlation was conducted to evaluate the relationship between haematological parameters and sleep quality scores. There is a significant negative relationship between RBC, Hb, and HCT levels and sleep quality (r_RBC_ = −0.157 p_RBC_ = 0.026; r_Hb_ = −0.158 p_Hb_ = 0.025; r_HCT_ = −0.175 p_HCT_ = 0.013). Such results suggest that as sleep quality worsens (indicated by higher scores), there is a tendency for haematological levels to decrease. However, a Wilcoxon-Mann-Whitney test was performed to evaluate whether firefighters with poor sleep quality presented lower haematological levels than firefighters with good sleep quality, with non-significant results (U_RBC_ = 4518.5; p_RBC_ = 0.157; U_Hb_ = 4522.5; p_Hb_ = 0.159; U_HCT_ = 4461.5; p_HCT_ = 0.125). Figure 1 illustrates the distribution of haematological levels in both good and bad sleeper groups.

To obtain a comprehensive insight into the observed changes in RBC, Hb, and HCT, it was crucial to consider other variables that could confuse the relationship between sleep quality and haematological parameters. Social demographic variables (age and sex) and behavioural groups, including smoking status (smokers vs. non-smokers), body weight (average weight vs. overweight), alcohol and coffee consumption habits (daily consumers vs. non-consumers), and engagement in regular physical exercise, were tested to evaluate if reported haematological profile differences between good and poor sleepers are due to sleep and not to covariable influence.

The results of the present study found sex to have a significant influence on haematological parameters, with males presenting higher blood levels (U_RBC_ = 817.000; p_RBC_ < 0.05; U_Hb_ = 534.5; p_Hb_ < 0.05; U_HCT_ = 534.5; p_HCT_ < 0.5); and better sleep quality than females (U_STS_ = 1686.0; p_STS_ ≤ 0.05). A negative correlation between sex and haematological levels was also found, indicating that, on average, males tend to have higher levels when compared to the opposite sex (r_RBC_ = −0.417; p_RBC_ < 0.05; r_Hb_ = −0.513; p_Hb_ < 0.05; r_HCT_ = −0.481; p_HCT_ < 0.05). Additionally, a positive correlation emerged between sex and sleep quality (r_STS_ = 0.187, p_STS_ = 0.013). Furthermore, a noteworthy trend was identified, as a decrease in sleep quality is associated with a reduction in blood levels. However, it is necessary to consider sex as a potential confounding factor in the relationship between sleep quality and haematological variables. Despite the initial correlations, when performing an ANCOVA analysis test, controlling for covariable Sex did not reveal a significant effect of quality of sleep on haematological levels ((F_RBC_(1,161) = 0.59, *p* = 0.88); (F_Hb_(1,176) = 0.00, *p* = 0.99); (F_HCT_(16,161) = 0.59, *p* = 0.89)). The relationship between haematological levels and age did not reach statistical significance (*p* > 0.05). A tendency towards an excess of weight in the sample of firefighters was observed, and a positive correlation was found between haematological levels and BMI (r_RBC_ = 0.145; p_RBC_ < 0.05; r_Hb_ = −0.172; p_Hb_ < 0.05; r_HCT_ = 0.142; p_HCT_ < 0.5), suggesting that blood levels increase with higher BMI values. Body mass index was also found to have a significant influence on haematological parameters, with excess-weight individuals presenting higher blood levels, indicating potential physiological changes related to body composition and metabolic factors [42,43,44] (U_RBC_ = 2836.5; p_RBC_ < 0.05; U_Hb_ = 3115.5; p_Hb_ < 05; U_HCT_ = 3043.5; p_HCT_ < 0.5). The ANCOVA analysis test, controlling for covariable BMI, did not reveal a significant effect of quality of sleep on haematological levels ((F_RBC_(1,186) = 1.60, *p* = 0.21); (F_Hb_(1,186) = 0.89, *p* = 0.344); (F_HCT_(1,186) = 1.427, *p* = 0.23)). When assessing the impact of exercise habits on haematological levels, statistical tests showed no correlation or significant difference in haematological levels between individuals who exercise and those who do not (*p* > 0.05).

Following the examination, results concerning blood levels within a group of smokers and non-smokers and groups of alcohol consumption were found to be statistically non-significant (*p* > 0.05).

Examining the potential impact of coffee on haematological parameters, our findings indicate a negative correlation between variables (r_RBC_ = −0.145; p_RBC_ < 0.05; r_Hb_ = −0.191; p_Hb_ < 0.05; r_HCT_ = −0.146; p_HCT_ < 0.5), indicating that coffee consumers present lower haematological levels than non-consumers (U_RBC_ = 1657.0; p_RBC_ < 0.05; U_Hb_ = 1483.0; p_Hb_ < 0.05; U_HCT_ = 1651.5; p_HCT_ < 0.5). However, non-parametric ANCOVA results suggest insignificant differences in haematological levels after adjusting for the effect of coffee consumption ((F_RBC_(1,186) = 1.60, *p* = 0.21); (F_Hb_(1,193) = 0.329, *p* = 0.25); (F_HCT_(1,192) = 1.651, *p* = 0.200)).

In our exploration, a multiple linear regression was used to understand how QS and the various confounding variables can impact haematological levels (RBC, Hb, and HCT) (Supplementary Material). The model for RBC was statistically significant, as indicated by the F-statistic of 6.053, with a *p*-value less than 0.001 (F(8,136) = 6.053, *p <* 0.001), suggesting that the model explains a significant portion of the variation in RBC levels. The adjusted R^2^ value of 0.219 illustrates that including variables explains 21.9% of the RBC variation. Gender (β = −0.428, *p <* 0.001), Age (β = −0.007, *p* = 0.036), and BMI (β = 0.152, *p* = 0.046) were the only significant predictors of red blood cell count (RBC). Sleep quality, exercise, coffee consumption, alcohol consumption, and smoking were not significant predictors of red blood cell count (RBC) (*p* > 0.05). The linear regression analysis for Hb was also statistically significant (F(8,139) = 8.375, *p <* 0.001), suggesting that the model explains a significant portion of Hb variance levels. The adjusted R^2^ value of 0.286 illustrates that our model can account for approximately 28.6% of Hb variability, highlighting the included predictors’ substantial impact. Gender (β = 0.552, *p <* 0.001) was the only significant predictor of Hb count. The linear regression analysis for HCT was also statistically significant (F(8,136) = 8.854, *p <* 0.001), suggesting that the model explains a significant portion of HCT variance levels. The adjusted R^2^ value of 0.304 illustrates that our model can account for approximately 30.4% of HCT variability, highlighting the included predictors’ substantial impact. Gender (β = −0.533, *p <* 0.001) and smoking (β = −0.145, *p* > 0.05) were the only significant predictors of Hb count.

To visually demonstrate the relationship between sleep quality and haematological parameters, we have drawn regression lines while accounting for the significant variables identified in the study (Sex, BMI, and Coffee).

**Figure 2:**
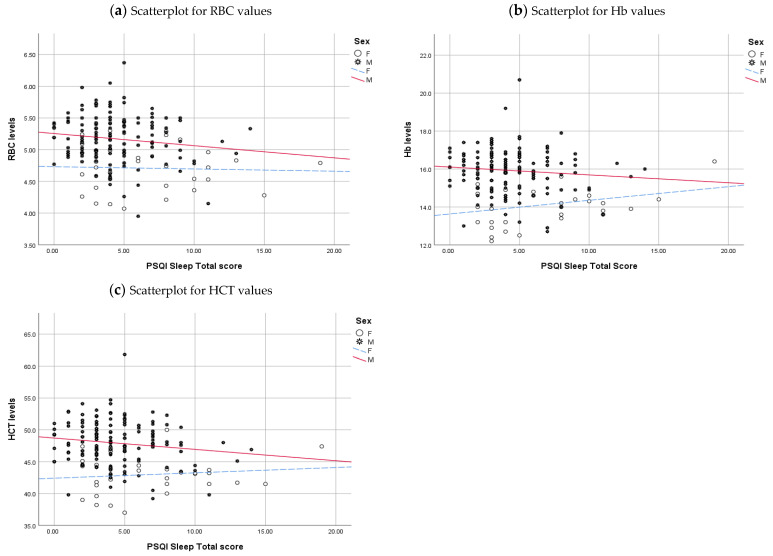
Scatterplot with regression lines representing the relationship between haematology parameters, PSQI Sleep Quality Total Score, and sex. Haematologial values in the Y-axis and PSQI sleep total scores, ranging from 0 to 21 in the X-axis. Males are in the star and solid red regression lines; females are in open circles and dashed blue regression lines.

The correlation between sleep scores and sex (Figure 2) exhibits a modest impact, explaining a mere 0.2% variability in RBC levels. However, this association becomes more notable within the male demographic, contributing 1.9% to the variance in RBC. In contrast, the connection is weaker for females, exposing 1.4% of the variability in RBC. This linear relationship extends its influence to 4.7% of the variability in Hb levels. Intriguingly, this connection is notably robust among females, revealing 8.8% of the variability in Hb. At the same time, it registers a more subdued impact for males, contributing a mere 1.0% to the variability in Hb. The linear relationship also extends to HCT, accounting for about 3.6% of the variability. This connection is less noticeable among females, explaining 1.5% of the variability in HCT. Conversely, for males, the relationship strengthens slightly, contributing 1.9% to the variability in HCT.

**Figure 3:**
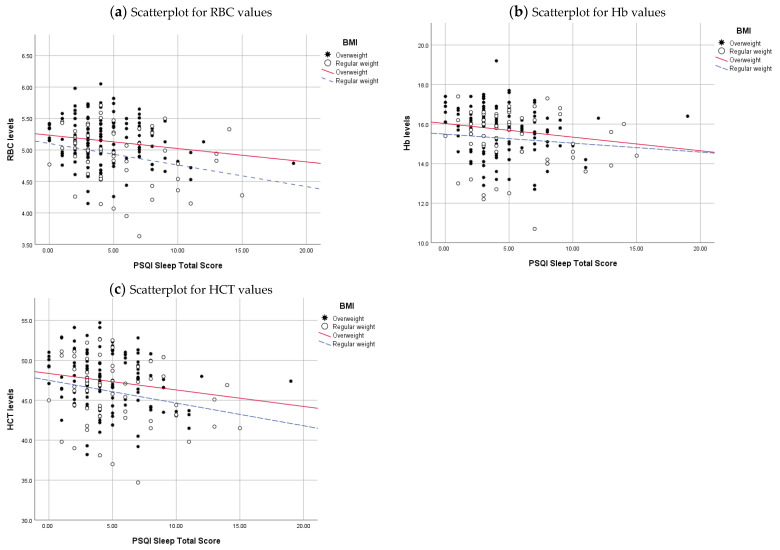
Linear regression lines represent the relationship between Haematology parameters, PSQI Sleep Total Scores, and BMI. Haematologial values in the Y-axis and PSQI sleep total scores, ranging from 0 to 21 in the X-axis. Excess weight is represented by a star and a solid red regression line, and regular weight is represented by open circles and a dashed blue regression line.

Figure 3 showcases the relationship between haemoglobin (Hb) and sleep total scores, coupled with BMI, revealing that this linear connection accounts for 5.1% of the variability in Hb (R^2^ = 0.051). Among individuals classified as overweight, the linear relationship contributes 2.8% to the variability in Hb. Similarly, within regular-weight individuals, the linear relationship explains 5.1% of the variability in Hb. Regarding haematocrit (HCT), approximately 4.7% of the variability is attributed to the linear relationship between sleep total scores and BMI. For those classified as overweight, this linear relationship explains 3.1% of the variability in HCT, while for regular-weight individuals, it contributes to 5.5%. These findings highlight the nuanced impact of sleep and BMI on haematology parameters, revealing distinct patterns among overweight and regular-weight individuals.

**Figure 4:**
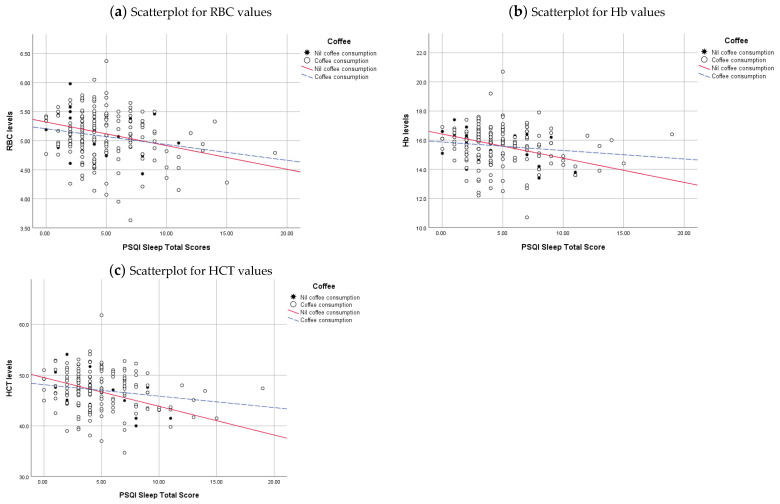
Linear regression lines represent the relationship between Haematology parameters, PSQI sleep total scores and coffee intake. Haematologial values in the Y-axis and PSQI sleep total scores, ranging from 0 to 21 in the X-axis. Nil coffee intake is represented by a star and a solid red regression line, and coffee consumption is represented by open circles and a dashed blue regression line.

When illustrating the relationship through regression lines to visually showcase the relationship between haematology parameters, sleep quality, and coffee consumption. The RBC overall relationship was an R^2^ linear of 0.045, signifying that approximately 4.5% of the variability can be explained by the linear connection with sleep quality (Figure 4). This connection intensifies among individuals not consuming coffee, clarifying 10.1% of the variability. On the contrary, the link is undermined for coffee consumers, explaining 3.8% of the variability. The linear relationship with sleep quality explains 2.8% of the variability in Hb. Among those refraining from coffee, this relationship amplifies, revealing 19.9% of the variability, whereas it diminishes to 1.8% for coffee consumers. Approximately 4.5% of the variability in HCT can be explained by the linear relationship with sleep. The relationship strengthens significantly to 25.5% for individuals with no coffee consumption. In contrast, for coffee consumers, the link weakens to 3.1%.

## 3. Discussion

The present study investigated the relationship between sleep quality and haematological parameters among firefighters in Northeast Portugal. Our results indicated a negative association between sleep quality and haematological levels, suggesting that deteriorating sleep quality correlates with altered haematological levels. Despite most firefighters presenting haematological profiles within reference ranges, a trend toward lower levels was observed. Additionally, sex significantly influenced haematological parameters, with males generally exhibiting higher blood levels and better sleep quality than females.Adequate sleep is crucial for firefighters, as it directly impacts their job performance and safety. Research indicated that insufficient sleep could lead to workplace mistakes, reduced alertness, lower productivity, and an increased risk of accidents, jeopardising their well-being and the safety of others [45]. 

The Sleep Research Society recommends that adults aim for 7–9 h of sleep per night for optimal health [46]. Disruptions to this essential biological need can significantly impact various aspects of health [45,46,47]. Recent epidemiological studies have revealed that sleep deprivation can lead to physiological changes that increase the risk of chronic diseases [41,42,43], including hypertension [41,44,48] (HT), coronary heart disease [48,49,50,51,52] (CHD), and diabetes mellitus (DM) [53,54,55,56]. Meeting optimal levels of sleep was proven to have survival benefits and reduce the risk of all-cause mortality while, in turn, providing a better quality of life [43,57]. There appears to be no effective self-perception of sleep quality [58,59], as individuals’ characterization of sleep quality is completely out of line with the assessment obtained by the PSQI [60]. This may be related to the fact that our sample is mostly male individuals who sometimes do not reveal or clearly express their feelings or feelings about their profession and its duties and generally have a wrong self-perception of their health quality [61,62,63]. It is important to emphasise that the data from our sample aligns with findings from a Portuguese study focusing on sleep habits. The referred study highlights the trend towards unsatisfactory and poor sleep quality among the general population. It suggests that sleep-related challenges are not exclusive to occupational settings but might reflect a larger societal issue regarding sleep health. These results indicate that, from a haematological perspective, the study population does not exhibit any significant abnormalities or deviations from the normal reference ranges, according to the Direção-Geral de Saúde, Regulation nº 21/2008. Similar to our study, Smith et al. (2001) found comparable results in the United States. They studied 11 male firefighters exposed to fire simulations, revealing an average of 4.94 (10^12^/L) erythrocytes, 14.3 g/dL haemoglobin, and 43.2% haematocrit [64]. These values fell within the established reference range for the U.S. population [64,65]. Likewise, Hunter et al. (2017) obtained similar outcomes. Their study of 19 healthy firefighters from Scotland during routine workdays showed haematological values within the normal range (Hb = 138 g/L; HCT = 0.40) based on reference values established in England [66]. Both studies faced limitations due to their small sample sizes, impacting the overall representativeness of their findings. Despite our study not having a notably larger sample size, it enabled us to derive more credible and statistically robust results. While there is existing research on firefighting and haematological values, there is a noticeable lack of scientific evidence regarding how shift work specifically affects haematological profiles among firefighters [65,66]. Most literature focuses on professions, such as the metallurgical industry, administrative roles, and healthcare, presenting altered haematological profiles— such as increased erythrocytes, Hb, and HCT—in individuals working extended or rotating work hours with a possible impact on sleep quality [67,68]. In particular, the studies conducted by Yen et al. (2019) [68] investigated the impact of shift work on haematological values across various professions, revealing higher Hb levels (15.36 g/dL) among night workers compared to those with daytime shifts [68]. Our results contribute to the findings of Wang et al.’, (2020) [69] research, which suggests that poor sleep may interfere with blood levels. Sleep disruption, although not entirely understood, can inhibit or suppress erythropoiesis [70]. Such disruption in the production of erythrocytes promotes a reduction in Hb concentration. Consequently, the decrease in Hb directly affects HCT levels, representing the proportion of RBC in the total blood volume [42,71,72,73,74]. Recent studies have also demonstrated that sleep supports the maintenance of haematopoiesis by calibrating the haematopoietic epigenome, curbing genetic drift, and maintaining clonal diversity [75]. Furthermore, the process of haematopoiesis follows a daily pace, with cell proliferation and function exhibiting variations based on the circadian rhythm. Specific cell types such as myeloid progenitor cells, leukocytes, and endothelial progenitors exhibit diurnal fluctuations, with their highest levels occurring during daylight hours. This pattern also influences the release of haematopoietic stem and progenitor cells and the production of peripheral blood leukocytes, both of which follow circadian patterns [76]. Diminished levels of Hb and HCT, resulting from poor sleep quality and the associated inhibition of erythropoiesis, can potentially contribute to the appearance of haematological pathologies such as anemia [42,77,78]. The correlation found in our research between higher sleep scores (reflecting poorer sleep quality) and diminished blood levels underscores the potential systemic impact of sleep on blood composition.

Our findings emphasise the nuanced interplay between sleep scores, sex, and haematological parameters. One potential explanation for such variability in results could be the smaller sample size for females, which may introduce variability and contribute to the observed positive correlation. Numerous studies on mammals consistently demonstrate higher levels of Hb and HCT in male adults than in female adults [79]. Such a difference is likely attributed to the stimulatory effect of androgens in males and the inhibitory effect of estrogen in bone marrow in females [25,79]. In addition, according to the scientific evidence, biological events inherent to women’s biology, such as the various hormonal activities present from birth to adulthood [80,81], from the female reproductive cycle throughout adolescence, pregnancy, and menopause, negatively affect the quality of female sleep [82,83]. As such, women are more prone to developing sleep disorders, namely obstructive sleep apnea, insomnia, narcolepsy, and restless legs syndrome [84]. Moreover, the use of hormonal contraceptives such as contraceptive pills is also a factor that can influence sleep architecture [81,82,84,85,86,87]. Further research may offer deeper insights into the complexities of the relationship between sleep quality, sex, and haematological parameters in females. Although the relationship between haematological levels and age did not reach statistical significance, some studies, such as those carried out by Ahmad et al. (2020) [88] and Cho et al. (2020) [89] in children and elderly samples, respectively, showed differences in haematological values according to age [88,89]. Additionally, further research has demonstrated that Hb and HCT values tend to decrease with age [89,90,91]. Several factors have been identified as influencing blood counts in older people. These include diminished counts of haematopoietic stem cells, the limited number of cell divisions [92], impaired proliferation of progenitor cells [93], inadequate mobilisation of such progenitors [94], and a deficiency in hormonal stimulation or diminished response to hormonal stimulation [95]. Even though the scientific community is still discussing a possible modification of haematological reference values based on age, once it is already accepted that lower blood results are a physiological phenomenon between older people [96,97,98].

Regarding BMI, research typically explores the correlation between BMI and sleep in the context of disorders or altered health states; there is a scarcity of studies examining the haematological profile of healthy adults about BMI [99,100]. Being associated with chronic inflammation, excess weight promotes atherosclerosis and metabolic syndrome [101,102], the precursors of a wide range of metabolic complications, such as diabetes and cardiovascular diseases [103]. Excess weight is also associated with hypertension, which is associated with higher RBC levels, increased Hb, and elevated HCT [104]. Our results suggest that blood levels increase with higher BMI values. As Jeong et al. (2021) [105] observed in a sample of 7950 individuals from Korea, there is a direct relationship between BMI and Hb/HCT values in both male (4229) and female (3721) individuals. Three BMI groups were distinguished (average weight, overweight, and obesity), showing the same pattern in both genders [105]. In males, Hb values were 14.57 g/dL, 14.61 g/dL, and 14.78 g/dL, respectively, and HCT values were 43.28%, 43.58%, and 44.09%, respectively. For females, the standard weight group had an average Hb of 13.27 g/dL and an average HCT of 40.05%. The overweight group had an average Hb of 13.30 g/dL and an average HCT of 40.25%, while the obese group showed an average Hb of 13.27 g/dL and an HCT of 40.34%. It is evident that as BMI increased, haematological values also tended to rise.

The present study did not find any correlations with the exercise variable. These results highlight the need for more investigation into such a field of interest, especially considering that other research indicates that exercise can cause an increment in blood levels as an adaptation response to training [42,106,107]. Such variations and mechanisms are only partially understood [108,109]. Exercise can stimulate the creation of new red blood cells. Paradoxically, exercise can also lead to decreased RBC due to intravascular haemolysis, which is associated with the rupture of red blood cells as they go through capillaries during muscle contraction. Additionally, the compression of red blood cells contributes to their breakdown, influencing the overall red blood cell mass [108]. The variability in HCT levels according to exercise practice is evident in studies. However, some reported lower levels [108,109,110], explained by the effect of auto-hemodilution from plasma volume expansion in response to exercise stimulus [111]. Others indicated higher-than-average values [112] due to diminished plasma volume, higher blood pressure, dehydration, sweating, and alterations in plasma water [113]. The differences may be attributed to the nature of firefighters’ work, which involves fluctuating intensity levels. Firefighters often experience periods of low activity, punctuated with intense activity, during emergency response; for this study, blood samples were collected during moments of rest, which could contribute to the observed results. Although our study did not observe a relationship between smoking and haemoglobin, it would be expected, as Hb and HCT are intrinsically and mutually dependent. Scientific literature indicates that tobacco consumption affects haematological values, causing a rise in Hb and HCT levels [114] due to the effects of nicotine and carbon monoxide [115,116]. Carbon monoxide, present in tobacco, causes a chemical reaction resulting in the formation of a new protein-carboxyhaemoglobin. The production of this new protein impairs red blood cells from functioning correctly, making it difficult for them to transport oxygen. In response to this situation, the body compensates for the lack of oxygen by entering a state of hypoxia, leading to a chemical response by the brain to stimulate red blood cell production, thereby increasing Hb and HCT levels [117,118]. Aldosari et al. (2020) presented similar results, finding that Hb and RBC levels hardly differed between the two groups [117]. Smokers had an average Hb level of 15.84 g/dL and a value of 5.68 (×10^12^/L) for red blood cells. On the other hand, non-smokers had an average Hb level of 15.70 g/dL and 5.58 (×10^12^/L) for RBC. Additionally, it was observed that smokers had a higher quantity of macrocytic red blood cells compared to non-smokers, indicating a larger mean corpuscular volume in individuals who smoked. This study corroborates our data in a specific aspect, as no association was found between tobacco consumption and Hb and RBC levels. However, Ahmed et al. (2024) [118] presented different results from the previous study. Aiming at evaluating the relationship between tobacco smoking and haematological parameters among 120 Sudanese healthy smokers, it revealed that smokers had a significantly higher RBC count of 5.37  ±  0.42 (×1012/L) and Hb levels of 16.17  ±  1.58 (10^12^/L). 

It is known that alcohol consumption can influence Hb and HCT levels [119] by suppressing blood cell production, or haematopoiesis. This impairment typically becomes significant in individuals with severe alcoholism due to the dose-dependent toxic effects of alcohol. Chronic, excessive alcohol consumption reduces the count of blood cell precursors in the bone marrow. It leads to distinct structural abnormalities in these cells, decreasing the number of mature and functional blood cells. Consequently, individuals with alcoholism may experience moderate anemia [119].

Alcohol consumption in the present study revealed no significant results, contrary to Kelley et al. (2021) [120], who analysed 2167 individuals, studying blood alcohol concentration and its correlation with haematological values across four groups: Group 1 (<0.10 g/dL), Group 2 (0.10–0.15 g/dL), Group 3 (0.15–0.20 g/dL), and Group 4 (>0.20 g/dL). Mean Hb values slightly decreased from the first to the second group, followed by a continuous increase. Mean Hb values exhibited a similar pattern. These results, inconsistent across alcohol concentrations, align with Jain et al.’ (2020) findings, suggesting varying effects of alcohol on Hb and its potential to inhibit haematopoiesis, leading to conditions like anemia [121].

The influence of coffee on haematological values remains conclusive based on the available data. The scarcity of research in this area underscores the necessity for further studies to elucidate the potential effects of excessive coffee and caffeine consumption on haematological parameters, providing crucial insights to the broader population. Regarding our study’s outcomes, the observed trends towards decreased haemoglobin and haematocrit may have arisen by chance. While our sample indicated this decline, larger-scale studies or investigations involving diverse populations are imperative to validate or refute this observation.

## 4. Materials and Methods

### 4.1. Design

The present study follows a cross-sectional observational design developed between June 2021 and September 2022 in fire stations in the Northeast region of Portugal.

### 4.2. Study Procedures

The data collection process was a wide-ranging procedure, conducted exclusively during the morning period, that incorporated two key components: a two-part questionnaire and blood sample collection. The study exclusively enrolled active-duty firefighters who had participated in firefighting activities, particularly forest fires, within the previous 24 to 48 h. Stringent adherence to the Declaration of Helsinki Oviedo Convention was ensured throughout the research process (approved protocol—Report Nr. 92/CEUP/2020—by the Ethics Committee of the University of Porto).

Research with human participants can only be conducted by ensuring and obtaining informed consent before data collection. As such, all firefighters aged 18 or older actively involved in forest fire response were provided with detailed information about the study objectives and procedures. They were then requested to present their informed consent by carefully reading and signing the form, thereby indicating their voluntary participation in the study. Submission to the National Commission for Data Protection (CNPN) was also accomplished. All personal data was stored separately from the participant’s name with an alphanumeric code number.

### 4.3. Questionnaire

The first part of the questionnaire was focused on gathering essential social, occupational, and demographic information from the participants. The second part applied the Portuguese version of the Pittsburgh Sleep Quality Index (PSQI), validated in 2017 [122], to access the self-reported quality of sleep (QS).

The Participant Profile Questionnaire gave a detailed understanding of the participants by capturing key variables for descriptive analysis. This comprehensive questionnaire effectively collected essential data such as age, sex, body mass index (BMI), smoking and exercise habits, and coffee and alcohol consumption. By incorporating these important variables, the questionnaire provided a robust foundation for conducting a thorough descriptive analysis, ensuring a comprehensive exploration of the participants’ characteristics and potential impact on the study outcomes.

The PSQI is organised into seven sections, with 19 questions rated 0 to 3, which evaluate different factors associated with sleep quality: Subjective sleep quality assesses an individual’s overall perception of their sleep quality; Sleep latency measures the time taken to fall asleep after getting into bed. Sleep duration evaluates the actual amount of time spent sleeping, considering both nighttime sleep and daytime napping. Sleep efficiency calculates the percentage of time spent asleep compared to the time spent in bed. Sleep disturbances assess the frequency of various sleep-related disturbances, such as difficulty falling asleep, waking up during the night, and having bad dreams. Use of Sleep medication investigates the use of sleep-inducing medications or substances and daytime dysfunction, which weighs the impact of sleep problems on daytime functioning, including difficulties with work, activities, and overall well-being [123].

The global PSQI score is the sum of all components, ranging between 0 and 21, with scores >5 indicative of worse sleep quality and suggestive of complications in some sleeping areas [123,124].

### 4.4. Blood Collection and Hemogram Profile Assessment

All blood samples were collected in the morning while fasting, following the recommendations for bood practice in phlebotomy [125]. Blood samples were collected and placed into the EDTA tube for a complete hemogram examination, executed following WHO guidelines on drawing blood [126]. Samples were correctly and anonymously labelled and placed in a correspondent rack for transportation. Transport was operated as requested by WHO specifications (2002) [127].

Shipment and manipulation of samples were realised on the same collection day, with a maximum of 4 h after venipuncture. A complete hemogram was performed by the automated haematology analyser PentraES60 (Horiba Medical Diagnostics, Montpellier, France), which is specific for clinical research. The haematological parameters analysed were as follows: red blood count (RBC), haemoglobin (Hb), haematocrit (HCT), mean corpuscular volume (MCV), mean corpuscular haemoglobin (MCH), mean corpuscular haemoglobin concentration (MCHC), red cell distribution width (RDW), platelet count and mean platelet volume (MPV), plateletcrit (PCT), and platelet distribution width (PDW).

### 4.5. Statistical Analysis

Descriptive statistics were employed to summarise the global characteristics of the sample. Sociodemographic variables (age and sex) and behavioural groups: smoking status (smokers vs. non-smokers), body weight (average weight vs. overweight), engagement in regular physical exercise, alcohol consumption habits (daily consumers vs. non-consumers), and coffee daily intake were included to assess their potential influence on the observed relationship. Data normality distribution was evaluated using tests of normality (Shapiro-Wilk and Kolmogorov-Smirnov), indicating not normally distributed data (*p* < 0.05).

To measure the strength and direction of the relationship between the variables of sleep quality and haematological profile, the non-parametric test Wilcoxon-Mann-Whitney and Spearman’s correlation coefficients were used. The effect of co-variables on haematological parameters was evaluated by applying the non-parametric ANCOVA test (Quade’s). In order to test the research question, a multiple linear regression was conducted, with age, sex (0 = male, 1 = female), smoking status (0 = smokers, 1 = non-smokers), body weight (0 = overweight, 1 = average weight), engagement in regular physical exercise (0 = nil exercise, 1 = daily exercise), alcohol consumption habits (0 = daily consumers, 1 = non-consumers), coffee daily intake (0 = daily consumers, 1 = non-consumers), and quality of sleep (poor sleep = 0, good sleeper = 1) as the predictors, with haematological levels as the dependent variable. Despite observing deviations from normality in the raw data, the assumptions of linearity, independence, homoscedasticity, and normality of residuals necessary for linear modelling were met. This confirmation allowed us to proceed with subsequent analysis using linear regression techniques. Moreover, the robustness of linear modelling to deviations from normality is supported by the substantial sample size in our study, which includes over 200 individuals. This large dataset provides a sufficient volume of data, which can enhance the resilience of linear modeling to deviations from normality. Statistical analysis was conducted using IBM SPSS software, version 26.0 (IBM Corp., Armonk, NY, USA) for a α = 0.05.

## 5. Conclusions

While our sample’s haematological parameters remain within the normal range, it is vital to recognise that they are not immune to the potential health consequences of sleep disruption, revealing a tendency for decreased blood levels in situations of poor sleep quality. Such conditions can contribute to the development of haematological disorders in the future. The limited scientific research in this field and the possibility of a chance correlation make it challenging to establish a strong association between sleep quality and haematological values. Future research with larger sample sizes and alternative statistical methods is necessary to explore further and understand the relationship between sleep and haematological parameters, which could potentially guide health interventions and screenings for firefighters, improving their well-being and safety. Furthermore, related research can be used to raise public awareness regarding the importance of sleep for overall health.

## Figures and Tables

**Table 1:** Sample characterization.

Sociodemographic Variables		*n*	%
Sex	Female	36	16%
	Male	170	73%
	No answer	27	12%
	Total	233	100%
Age group	18–34	89	38%
	35–49	107	46%
	>50	30	13%
	No answer	7	3%
	Total	233	100%
BMI	Underweight (≤16–18.49 kg/m^2^)	3	1%
	Average weight (18.50–24.99 kg/m^2^)	62	27%
	Pre-obesity (25.00–29.99 kg/m^2^)	102	44%
	Obesity I (30.00–34.99 kg/m^2^)	38	16%
	Obesity II (35.00–39.99 kg/m^2^)	8	3%
	Obesity III (≥40.00 kg/m^2^)	3	1%
	No answer	17	7%
	Total	233	100%
Physical exercise	Yes	172	74%
	No	54	23%
	No answer	7	3%
	Total	233	100%
Tobacco Consumptions	Smoker	93	40%
	Nonsmoker	135	58%
	No answer	5	2%
	Total	233	100%
Alcohol intake	Consumer	58	25%
	Yes	156	67%
	No	19	8%
	Total	233	100%
Coffee Habits	Yes	192	82%
	No	31	13%
	No answer	10	4%
	Total	233	100%

**Table 2:** Sleep quality characterization.

PSQI Dimensions		%	Mean ± SD
PSQI Subjective Quality of Sleep	Very good	30	0.83 ± 0.58
	Good	59	
	Bad	8	
	Very bad	4	
	Total	100	
PSQI Sleep latency	≤15 min	31	1.13 ± 0.86
	16 to 30 min	38	
	31 to 60 min	18	
	≥60 min	14	
	Total	100	
PSQI Sleep duration	More than 7 h	39	0.89 ± 0.86
	6 to 7 h	34	
	5 to 6 h	24	
	Less than 5 h	4	
	Total	100	
PSQI Sleep efficiency	Above 85%	74	0.42 ± 0.84
	75% to 84%	15	
	65% to 74%	5	
	Under 65%	6	
	Total	100	
PSQI Sleep disturbance	Nule disturbance	10	1.03 ± 0
	Rare disturbance	78	
	Few disturbance	13	
	Many disturbance	0	
	Total	100	
PSQI Use of sleep medication	Never	88	0.17 ± 0.57
	Less than once a week	7	
	1 to 2 times a week	1	
	≥3 times a week	3	
	Total	100	
PSQI Daytime disfunction	No disfunction	100	0.29 ± 0.58
	Rare disfunction	74	
	Some disfunction	20	
	Few disfunction	6	
	Many disfunction	1	
	Total	100	
PSQI global score	Good sleeper (PSQI < 5)	55	4.74 ± 3.05
	Poor sleeper (PSQI > 5)	45	
	Total	100	

## Data Availability

The original contributions presented in the study are included in the article/Supplementary Material, further inquiries can be directed to the corresponding author.

## Supplementary Materials

The following supporting information can be downloaded at: https://www.mdpi.com/article/10.3390/clockssleep6030021/s1. The results of the multiple regression analysis are showcased in a series of tables available as Supplementary Material (Supplementary Material S1).
