# Supplementary material for: The Impact of Sleep on Haematological Parameters in Firefighters
Source: Clocks Sleep. 2024 Jun 26;6(3):291–311. doi: 10.3390/clockssleep6030021 (PMC11270419; doi:10.3390/clockssleep6030021)
Supplement: Supplementary file 1 [file clockssleep-06-00021-s001.zip › clockssleep-2950366-supplementary.pdf]

Supplement

Results of the multiple regression analysis

RBC multiple regression analysis results

Supplemental table 1 RBC Model Summary

Model Summary	
Multiple R	.512
R^2	.263
Adjusted R^2	.219
Standard Error	.359

Supplemental table 2 RBC Analysis of variance

Analysis of variance

	Sum of Square	DF	Mean Square	F	Sig.
Regression	6.253	8	.782	6.053	.000
Residual	17.561	136	.129		
Total	23.814	144			

Supplemental table 3 RBC Coefficients: variables included in the equation

Variables included in the equation

	Unstandardized Coefficients		Standardized coefficients		
	B	Std error	Std Coefficients Beta	t	Sig.
SQ	.077	.062	.092	1.240	.217
Sex	-.428	.08	-405	-5.166	.000
BMI	.134	.067	.152	1.995	0.48
Exercise	.010	.030	.024	.319	.751
Coffee	-.152	.093	-.127	-.635	.104
Smoking	-.025	.062	-.031	-.406	.685
Drinking	.018	.066	.020	.273	.785

Hb multiple regression analysis results

Supplemental table 4 Hb Model Summary

Model Summary	
Multiple R	.570
R^2	.325
Adjusted R^2	.286
Standard Error	.9849

Supplemental table 5 Hb Analysis of variance

Analysis of variance

	Sum of Square	DF	Mean Square	F	Sig.
Regression	64.994	8	8.124	8.375	.000
Residual	134.833	139	.970		
Total	199.827	147			

Supplemental table 6 Hb Coefficients: variables included in the equation

Variables included in the equation

	Unstandardized Coefficients		Standardized coefficients		
	B	Std error	Std Coefficients Beta	t	Sig.
SQ	-.062	.259	-.027	-.241	.810
Sex	1.686	.237	.552	7.122	.000
BMI	-.036	.181	-.014	-.197	.844
Exercise	-.042	.194	-.015	-.216	.829
Coffee	-.515	.27	-.134	-1.866	.064
Smoking	.253	.165	.108	1.534	.127
Drinking	.036	.180	.014	.202	.844

<u>HCT multiple regression analysis results</u>

Supplemental table 7 HCT Model Summary

Model Summary	
Multiple R	.585
R^2	.342
Adjusted R^2	.304
Standard Error	2.975

Supplemental table 8 HCT Analysis of variance

Analysis of variance

	Sum of Square	DF	Mean Square	F	Sig.
Regression	626.903	8	78.363	8.854	.000
Residual	1203.691	136	8.851		
Total	1830.594	144			

Supplemental table 9 HCT Coefficients: variables included in the equation

Variables included in the equation

	Unstandardized Coefficients		Standardized coefficients		
	B	Std error	Std Coefficients Beta	t	Sig.
SQ	.613	.513	.084	1.195	.234
Sex	-4.935	.685	-.533	-7.201	.000
BMI	.439	.554	.057	.792	.430
Exercise	.249	.251	.070	.990	.324
Coffee	-.654	.770	-.062	-.849	.397
Smoking	-1.040	.517	-.145	-2.011	.046
Drinking	-.130	.548	-.017	-.238	.812
